# Immune and Clinical Features of *CD96* Expression in Glioma by *in silico* Analysis

**DOI:** 10.3389/fbioe.2020.00592

**Published:** 2020-06-30

**Authors:** Qiang Zhang, Hua Zhong, Yinchun Fan, Qian Liu, Jiancheng Song, Shengtao Yao, Fang Cao

**Affiliations:** ^1^Department of Cerebrovascular Disease, Affiliated Hospital of Zunyi Medical University, Guizhou, China; ^2^College of Life Sciences, Wuhan University, Wuhan, China

**Keywords:** *CD96*, glioma, immune checkpoint, prognosis, immunotherapy

## Abstract

**Background:**

Immune checkpoints target regulatory pathways in T cells that enhance antitumor immune responses and elicit durable clinical responses. As a novel immune checkpoint, *CD96* is an attractive key target for cancer immunotherapy. However, there has been no integrative investigation of *CD96* in glioma. Our study explored the relationship between *CD96* expression and clinical prognosis in glioma.

**Methods:**

RNA and clinical data for a total of 1,001 samples were included in this study, including 325 samples from the Chinese Glioma Genome Atlas (CGGA) database and 676 samples from The Cancer Genome Atlas (TCGA) dataset. The R programming language was employed to perform statistical analysis and draw figures.

**Results:**

*CD96* had a consistently positive relationship with glioblastoma and was highly enriched in IDH-wildtype and mesenchymal subtype glioma. Gene ontology enrichment and gene set variation analysis analyses suggested that *CD96* was mostly involved in immune functions and was especially related to T cell-mediated immune response in glioma. Subsequent immune infiltration analysis showed that *CD96* was positively correlated with infiltrating levels of CD4 + T and CD8 + T cells, macrophages, neutrophils, and DCs in glioblastoma multiforme and low-grade glioma. Additionally, *CD96* was tightly associated with other immune checkpoints, including *PD-1*, *CTLA-4*, *TIGIT*, and *TIM-3*. Univariate and multivariate Cox analysis demonstrated that *CD96* acts as an independent indicator of poor prognosis in glioma.

**Conclusion:**

*CD96* expression was increased in malignant phenotype and negatively associated with overall survival in glioma. *CD96* also showed a positive correlation with other immune checkpoints, immune response, and inflammatory activity. Our findings indicate that *CD96* is a promising clinical target for further immunotherapeutic use in glioma patients.

## Introduction

The most prevalent and devastating primary intracranial tumor, glioma, and especially glioblastoma multiforme (GBM, WHO grade IV), are characterized by heterogeneity and extensive invasion, with high recurrence and fatality rate (Jiang et al., 2016; Louis et al., 2016; Van Meir et al., 2010). Therefore, multiple attempts have been made to prolong life expectancies of glioma patients, comprising developing effective therapy, appropriate biomarkers, and molecular-targeted drugs. Numerous conventional treatment methods of central nervous system (CNS) tumors have emerged in recent decades, such as neurosurgical resection, radiotherapy, and chemotherapy (Stupp et al., 2009; Walker et al., 2017). Among them, immunotherapy is reckoned to be an encouraging treatment because it evokes an antitumor immune response to restrain immune evasion of the tumor (Louveau et al., 2015). In melanoma and non-small-cell lung cancer, immune checkpoint inhibitors like target programmed cell death protein 1 (*PD-1*)/programmed death ligand 1 (*PD-L1*) and cytotoxic T-lymphocyte-associated antigen-4 (*CTLA-4*) have been exploited and successfully applied in the clinic (Topalian et al., 2012; Wang et al., 2016; Huang et al., 2017), whereas many glioma patients are refractory to current immunotherapy, which arouses our interest in identifying additional immune checkpoints to enhance the therapeutic efficacy in glioma (Reardon et al., 2014; Huang et al., 2017).

A novel immune checkpoint receptor target, *CD96*, has recently entered the limelight in current cancer immunotherapies shown to inhibit natural killer (NK) cells (Georgiev et al., 2018). Compelling evidence confirmed that blocking *CD96* enhanced the containment of primary tumor growth in murine model systems in a CD8 + T cell-dependent manner (Dougall et al., 2017; Mittal et al., 2019). Of note, the efficiency of the antitumor activity of anti-*CD96* therapy improved in dual-combination with blockade of other immune checkpoints, like *PD-1*, *PD-L1, TIGIT*, and *CTLA-4* (Mittal et al., 2019). Besides, *CD96* represents several unique features that exhibit profound beneficial effects in the coming age of human cancer therapy (Georgiev et al., 2018; Deuss et al., 2019).

To make a systematic examination of *CD96* in glioma, we gathered RNA-seq and clinical data for 325 glioma samples from the Chinese Glioma Genome Atlas (CGGA) project. Simultaneously, we obtained another data set containing 676 samples from The Cancer Genome Atlas (TCGA) cohort to further corroborate the findings. Overall, our comprehensive study of the molecular and clinicopathological features of *CD96* through 1,001 samples will provide a better understanding of *CD96* in glioma and pave the way for developing *CD96*-targeted cancer immunotherapies.

## Materials and Methods

### Data Collection From the CGGA and TCGA Projects

The *CD96* genetic and clinical data of 325 glioma samples were downloaded from the CGGA cohort^1^; these ranged from WHO grade II–IV. The read counts for each GENCODE gene were calculated using RSEM and were transformed into a FPKM (fragments per kilobase transcriptome per million fragments) matrix. In this mRNA expression profile, an expressed gene was defined only if its expression level was larger than 0 in half of the samples.

Moreover, 161 GBM (glioblastoma multiforme) and 515 LGG (low-grade glioma) samples were obtained from the TCGA Pan-Cancer Atlas project through the cbioportal^2^ website^3^ including RNA-seq data and clinicopathological information. The read counts were also calculated using RSEM, and mRNA expression was batch corrected and normalized.

### Gene Ontology and Gene Set Variation Analysis Analysis

After Spearman correlation analysis, gene ontology (GO) analysis of the most correlated genes was constructed by using the R package ‘clusterProfiler’ (Yu et al., 2012). Gene set variation analysis (GSVA) analysis was applied using standard settings, as implemented in the R package ‘GSVA’ (Hänzelmann et al., 2013). Additionally, inflammatory-related metagenes were described previously (Wang et al., 2016).

### TIMER Database Analysis

The Tumor Immune Estimation Resource (TIMER) serves a comprehensive resource to analyze immune infiltrates among 10,897 samples across 32 cancer types (Li T. et al., 2017). The purity-corrected Spearman’s rho of *CD96* expression with the abundance of immune infiltrates, including B cells, CD4 + T cells, CD8 + T cells, neutrophils, macrophages, dendritic cells, T cell NK, and NK cells were detected in GBM and LGG patients on TIMER^4^ and with TIMER 2.0^5^ online tools.

### Statistical Computations

Statistical analysis and figure construction were conducted using the R language, version 3.6.1^6^. The *t*-test was performed to evaluate *CD96* expression among different grades, pathological subsets, IDH^*WT*^/IDH^*MUT*^, and subtypes of glioma. Kaplan–Meier plots and Cox proportional hazard model analysis were generated using the R packages ‘survminer’ and ‘survival’ (Therneau and Lumley, 2015; Kassambara et al., 2018). Receiver operating characteristic (ROC) curves were calculated by the R package ‘pROC’ (Robin et al., 2013). Area under the curve (AUC) values were depicted from the ROC curves. Corrgram and corrplot plots were drawn using the R packages ‘corrgram’ and ‘corrplot’, separately (Friendly, 2002; Wei, 2017). All statistical tests were two-sided, and a *p*-value < 0.05 was regarded as indicating statistical significance.

## Results

### CD96 Was Enriched in Glioblastoma, IDH-Wildtype, and Mesenchymal Glioma

**FIGURE 1:**
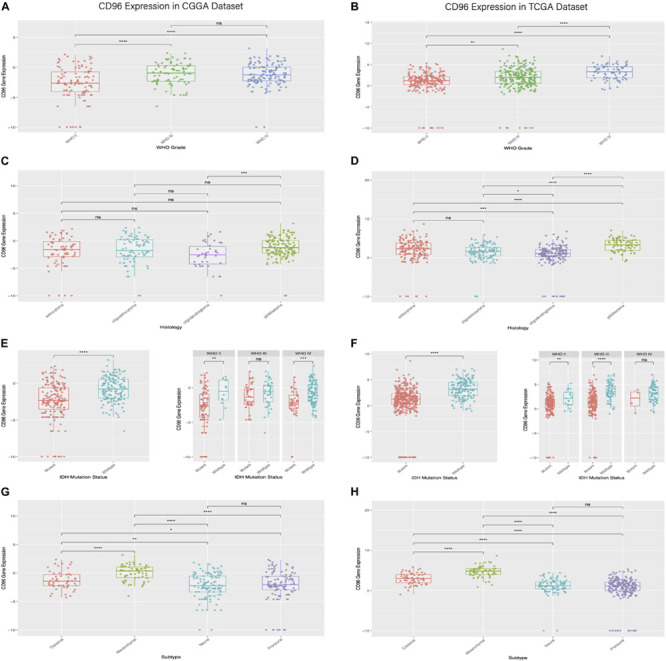
Relationship between CD96 expression and clinical glioma parameters in the CGGA and TCGA cohorts. **(A,B)** Correlation of *CD96* transcript levels and WHO grade. **(C,D)** *CD96* expression pattern in different WHO grades. **(E,F)** Association between *CD96* expression and IDH-wildtype for all-grade glioma. **(G,H)** Correlation of *CD96* transcript levels and glioma subtypes. Ns, **, ***, and **** represent no significant, *p* < 0.05, *p* < 0.01, and *p* < 0.0001, respectively.

To clarify differences in the *CD96* expression pattern in four grades of glioma malignancy, the mRNA level of *CD96* was examined in the CGGA and TCGA databases separately. In the CGGA cohort, the CD96 transcript profiles in WHO grade III and IV displayed little difference (not significant in *t*-test) and had higher values than WHO II (Figure 1A). In the TCGA dataset, *CD96* expression in glioblastoma (WHO IV) was higher than in WHO grade II and grade III glioma in TCGA (Figure 1B). The results revealed that *CD96* expression was comparatively upregulated in higher-grade gliomas.

The distribution of *CD96* in different glioma subtypes was next analyzed. Compared to other pathological subsets (namely oligodendroglioma, oligoastrocytoma, and astrocytoma), *CD96* expression was relatively upregulated in glioblastoma (Figures 1C,D). Isocitrate dehydrogenase (IDH) mutation is present in almost 40% of glioma, which has an outsized impact on glioma development and progression (Branzoli et al., 2018). To this end, the expression profile of *CD96* in different IDH states was also explored. We found that *CD96* was consistently enriched in IDH-wildtype (IDH^*WT*^) in both the CGGA and TCGA databases (Figures 1E,F). This result suggested that *CD96* expression was more prevalent without IDH mutation (IDH^*WT*^) than with IDH mutation (IDH^*MUT*^) in glioma. Besides, *CD96* was expressed at higher levels in the mesenchymal subtype compared with the other three subtypes (classical, neural, and proneural) in the CGGA cohort (Figure 1G). Highly consistent results were obtained using the TCGA dataset (Figure 1H).

### *CD96* Had Sufficient Sensitivity to Predict Mesenchymal Subtype Glioma in ROC Curve Analysis

**FIGURE 2:**
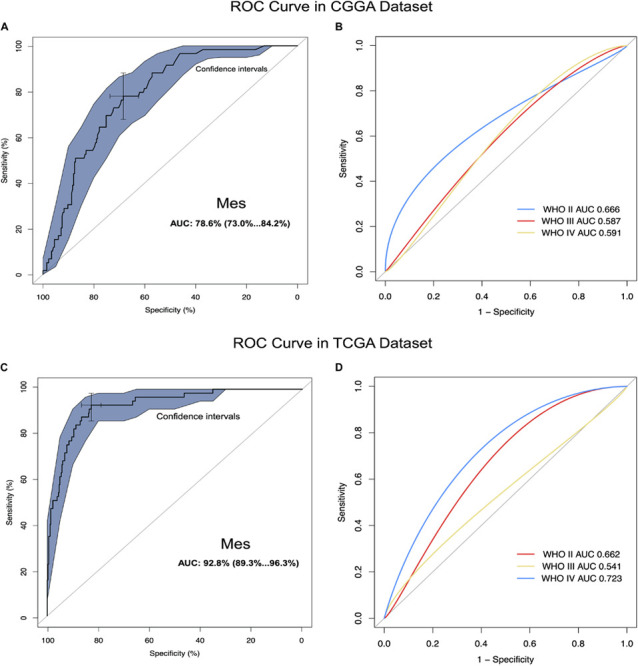
*CD96* was highly enriched in mesenchymal molecular subtype glioma. **(A,B)** ROC curve analysis of *CD96* in mesenchymal subtype and all-grade gliomas in the CGGA dataset. **(C,D)** ROC curve analysis of *CD96* in mesenchymal subtype and all-grade gliomas in the TCGA dataset.

We carried out ROC curves analysis of *CD96* expression and mesenchymal subtype in glioma. Intriguingly, we observed that the AUC values of ROC curves were up to 78.6. and 92.8% in the CGGA and TCGA datasets, respectively (Figures 2A,C). These findings indicated that *CD96* acted as a potential biomarker for mesenchymal subtype glioma. ROC curves analyses of *CD96* in glioma of all WHO grades were next conducted. The AUC of WHO IV was 0.723 in the CGGA database, whereas the AUC of WHO IV was 0.591 in the TCGA database (Figures 2B,D).

### CD96 Was Significantly Associated With Immune Functions in Glioma

**FIGURE 3:**
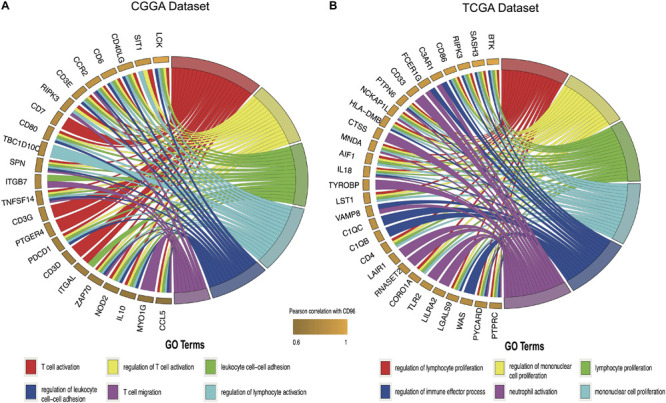
Gene ontology (GO) analysis for top 50 genes most relevant to *CD96* in the CGGA database **(A)** and TCGA database **(B)**.

**FIGURE 4:**
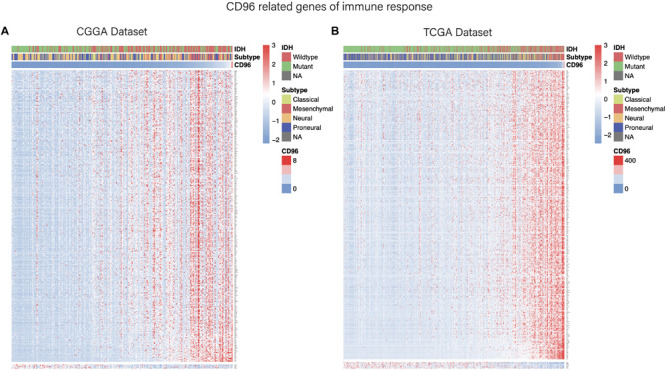
Heatmaps of CD96-related immune genes in glioma in the CGGA **(A)** and TCGA cohorts **(B)**.

Considering that *CD96* expression was closely tied to malignancy, we inferred that *CD96* became an integral part of glioma progression and therefore performed GO analysis to uncover its role. Spearman correlation analysis (Spearman | rho| > 0.6) indicated that 54 and 176 genes were positively correlated with *CD96* in the CGGA and TCGA datasets, respectively. According to GO biological process (BP) enrichment analysis for the top 50 related genes, we found that the genes most relevant to *CD96* were involved in T-cell activation and regulation of lymphocyte proliferation in the CGGA and TCGA databases, respectively (Figures 3A,B; Supplementary Table S1). To further elucidate the immune function of *CD96* in glioma, on the basis of the 1,540 genes reported to be associated with the immune response which were downloaded form the AmiGO 2 website^7^ (Li G. et al., 2017), we selected the 362 and 354 genes most relevant to *CD96* (Spearman |rho| > 0.3) in the CCGA and TCGA cohorts (Supplementary Table S2) for heatmap drawing (Liu et al., 2019). Thereinto, 357 genes were strongly positively correlated with *CD96* expression, while five genes had a significantly negative relationship with *CD96* in the CGGA dataset. In the TCGA dataset, 347 and 7 genes were directly and inversely proportional to *CD96* expression, respectively (Figure 4). To sum up, *CD96* was directly correlated with most immune responses and negatively correlated with few immune responses in glioma.

### Correlation Between *CD96* and T-Cell Mediated Immunity in Glioma

**FIGURE 5:**
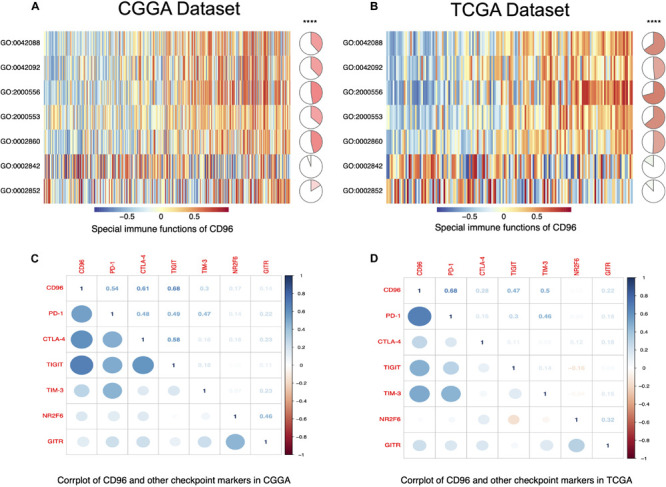
*CD96*-related T cell immunity and immune checkpoint markers in glioma. **(A,B)** The relationship between *CD96* and T cell immunity in glioma in the CGGA and TCGA datasets. **(C,D)** Corrgram map of CD68 and immune checkpoint markers in glioma in the CGGA and TCGA databases.

To fully understand the relationship between *CD96* and T cell-related immunity in glioma, we performed GSVA to assess differential activities of pathways between sets of genes. As delineated in Figures 5A,B, these relationships were similar in both the CGGA and TCGA databases. Specifically, *CD96* showed a positive correlation with T-helper 1/2 type immune response (GO:0042088 and GO:0042092), T-helper 1/2 cell cytokine production (GO:2000556 and GO:2000553), and NK cell-mediated cytotoxicity directed against tumor cell target (GO:0002860). Conversely, *CD96* was correlated negatively with T cell-mediated immune response to tumor cell (GO:0002842) and T cell-mediated cytotoxicity directed against tumor cell target (GO:0002852). This result re-validated that the special immune function of *CD96* is to act an inhibitory role in T cell-mediated immune response to tumor cells in glioma.

### Correlation Between *CD96* and Other Immune Checkpoints

A growing number of immune checkpoints have been emerged as therapeutic targets and have been examined in clinical trials or clinical situations (Ribas and Wolchok, 2018; Feng et al., 2019). Therefore, we analyzed the relationship between *CD96* and other immune checkpoints, such as *PD-1*, *CTLA-4*, *TIGIT*, *TIM-3*, *NR2F6*, and *GITR*. Pearson correlation analysis revealed that *CD96* was tightly associated with *PD-1*, *CTLA-4*, *TIGIT*, and *TIM-3*. Co-expression of *PD-1* with *CD96* was consistent with the findings of previous research (Mittal et al., 2019). These results were validated in both the CGGA and TCGA datasets (Figures 5C,D), implying possible synergistic effects of *CD96* with these checkpoint members. Accordingly, we postulated that *CD96* may contribute significantly to the inflammatory response in glioma and used a previously described method to test this (Wang et al., 2016). As shown in Supplementary Figure S1, *CD96* was positively associated with HCK, MHC-I, MHC-II, STAT1, STAT2, and FCGR2A, and especially with LCK. This finding additionally evidenced the vital immune function of *CD96* in glioma.

### Correlation Between *CD96* and Immune Infiltration Level in GBM and LGG

**FIGURE 6:**
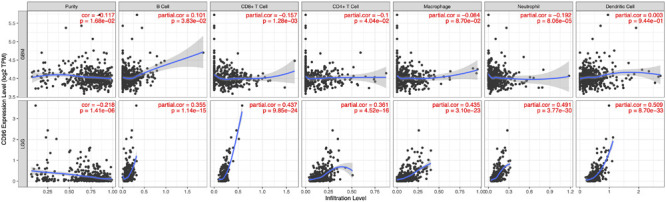
Correlation of *CD96* expression with immune infiltration level in GBM and LGG.

Tumor-infiltrating lymphocytes are an independent predictor of sentinel lymph node status and survival in cancers. Hence, we investigated whether *CD96* expression was connected with immune infiltration levels in GBM (glioblastoma multiforme) and LGG (Low-Grade Glioma) by using TIMER website tools. Our findings showed that *CD96* expression related positively with infiltrating levels of dendritic cells (*r* = 0.509), neutrophils (*r* = 0.491), macrophages (*r* = 0.435), and CD8 + T cells (*r* = 0.437) in LGG. Simultaneously, *CD96* had a marginal positively association with B cell (*r* = 0.355) and CD4 + T cell (*r* = 0.361) infiltration level in LGG. On the other hand, *CD96* expression has no significant relationships with tumor purity (*r* = -0.117) or infiltrating levels of B cells (*r* = 0.101), CD8 + T cells (*r* = -0.157), CD4 + T cells (*r* = -0.1), and other cells in GBM (Figure 6). These differences implied that *CD96* would make more of a contribution to immune infiltration in LGG than GBM, especially in dendritic cells. Using TIMER 2.0 tools, the associations between *CD96* expression and T cell NK and NK cell immune infiltrates were explored. As shown in Supplementary Figure S2, the *CD96* expression level showed a positive association with NK cell EPIC and a negative association with NK cell QUANTISEQ in both GBM and LGG. Additionally, *CD96* was positively correlated with NK cell activated CIBERSORT-ABS, while it was negatively correlated with NK cell XCELL. The low purity-adjusted Spearman’s rho (all rho <0.5) implied a weak correlation of *CD96* expression with the above immune infiltration levels (Figure 6 and Supplementary Figure S2). These findings could provide helpful information about the potential cellular targets of *CD96* blockade.

### *CD96* Predicted Worse Survival in Glioma

**FIGURE 7:**
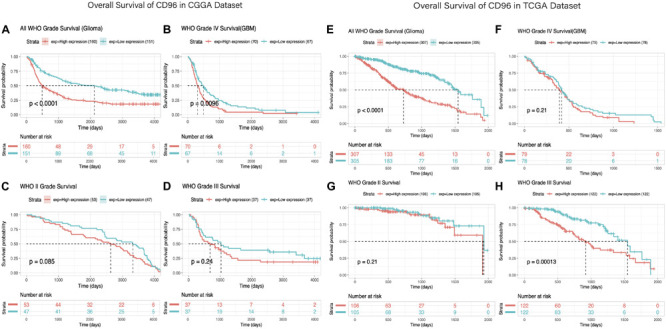
Survival analysis of glioma based on *CD96* expression. **(A–D)** Overall survival analysis of *CD96* in all-grade glioma and WHO IV, WHO II, and WHO III gliomas based on data from the CGGA cohort. **(E–H)** Overall survival analysis of *CD96* in all-grade glioma and WHO IV, WHO II, and WHO III gliomas based on data from the TCGA cohort. High and low expressions were defined as the CD96 transcript level being more or less than the median level of all samples.

Owing to the high relevance between *CD96* and immune suppressor in glioma, the prognostic impact of *CD96* was verified via the Kaplan–Meier method. Overall survival (OS) analysis in glioma patients demonstrated that high expression of *CD96* predicted relatively poor survival in the CGGA and TCGA cohorts (Figures 7A,E). Higher *CD96* expression is associated with worse OS in patients with WHO grades II, III, and IV glioma based on data from the CGGA dataset (Figures 7C,D,B, respectively). Similarly, as shown, strong associations were observed between higher expression of *CD96* and shorter OS for all WHO grades patients in the TCGA dataset (Figures 7F–H). These findings suggested that *CD96* is a negative prognostic indicator in glioma and GBM patients.

Eventually, we evaluated the independence of the clinicopathological significance of *CD96* in glioma by univariate and multivariate Cox regression analyses. The results indicated that *CD96*, age, gender, WHO grade, IDH mutation, and h1p19q codeletion status were closely associated with OS and revealed that *CD96* is an independent prognosticator for glioma patients (Supplementary Table S3).

## Discussion

Recent studies on tumor immunotherapy continue to show strong results and bring hope to glioma patients. Among those immunotherapeutic strategies, immune checkpoint blockade offers remarkable benefits to the therapies of various tumor types by increasing antitumor immunity (Postow et al., 2018). However, research in the field of neural tumor immunotherapy currently mainly focuses on *CTLA-4* and *PD-1*/*PD-L1* blockade. Nonetheless, potential immune-related adverse events hinder the wide clinical application of immunotherapy (Postow et al., 2018). The identification of alternative checkpoint targets may facilitate the solution of this predicament and bring new therapeutic benefits for cancer treatment.

Increasingly, *CD96* is emerging as a potent modulator of antitumor immune responses (Georgiev et al., 2018). *CD96* has been shown to negatively regulate NK cell-mediated immune surveillance and to intervene in multidimensional adhesion, inhibition, and activation of participating cells (Fuchs et al., 2004; Chan et al., 2014; Georgiev et al., 2018; Roman Aguilera et al., 2018). The special effect of *CD96* has also been reported in some tumors. Hepatocellular carcinoma (HCC) patients with high expression level of *CD96* within tumor are strongly correlated with deteriorating disease situations and shorter disease-free survival and OS times (Sun et al., 2019). Targeting host *CD96* appears to be an innovative strategy for clinical application in combination with current immunotherapies. Herein, we probed the biological functions of *CD96* in glioma through large scale and in-depth analyses. *CD96* expression was markedly enriched in higher-grade malignant pathological gliomas. Moreover, high expression of *CD96* was observed in the malignant molecule phenotype, including IDH wildtype and mesenchymal subtype. Collectively, *CD96* exhibited a malignant biological property in glioma. Meanwhile, through GO analysis of the relationship between *CD96* and BPs, we noted that *CD96* had a positive association with immune response and inflammatory activities.

A range of immunotherapies targeting checkpoint inhibitors and blocking monoclonal antibodies (mAbs) have been widely adopted, and several of them are ongoing in glioblastoma (Bouffet et al., 2016; Huang et al., 2017). Compared to monotherapy treatments, combination approaches were reported to be more effective and associated with substantially longer progression-free survival (Larkin et al., 2015; Postow et al., 2015). In our research, *CD96* showed a high concordance with immune checkpoints *PD-1*, *CTLA-4*, *TIGIT*, *TIM-3*, *NR2F6*, and *GITR*, indicating the potential synergistic effects of these markers. We inferred that collaboration of *CD96* with other checkpoint members, especially *PD-1*, may increase glioma immunotherapy effectiveness. Indeed, previous studies have demonstrated that concurrent blockade of *CD96* and *PD-1* increased antitumor immunity more than targeting *PD-1* alone, potentially without inducing serious 908 immune-related toxicities (Blake et al., 2016; Harjunpää et al., 2018). This provided support to our research. We also discovered that higher *CD96* expression predicted worse survival rates in glioma and GBM patients. This significant prognostic signature implied that *CD96* blockade may significantly improve the prognosis of glioma patients, especially GBM patients.

## Conclusion

To sum up, we initially explored the genetic and clinical characteristics of *CD96* based on CGGA and TCGA datasets. Our results highlighted that *CD96* may be a promising biomarker and therapeutic target for glioma that presents favorable application prospects.

## Data Availability Statement

Publicly available datasets were analyzed in this study. This data can be found here: http://cancergenome.nih.gov/ and http://www.cgga.org.cn/.

## Ethics Statement

All the procedures in this study were approved by the ethics committees of all hospitals, and written informed consent was obtained from all patients.

## Author Contributions

SY and FC conceptualized and designed this study. HZ performed the data collection and analysis. QZ wrote the manuscript. YF, QL, and JS participated in constructing figures and revision. All authors gave final approval of the manuscript.

## Conflict of Interest

The authors declare that the research was conducted in the absence of any commercial or financial relationships that could be construed as a potential conflict of interest.

## Footnotes

1 http://cgga.org.cn/

2 https://www.cbioportal.org

3 http://www.cbioportal.org/study/summary?id=gbm_tcga_pan_can_atlas_2018; http://www.cbioportal.org/study/clinicalData?id=lgg_tcga_pan_can_atlas_2018

4 https://cistrome.shinyapps.io/timer

5 http://timer.cistrome.org/#

6 http://www.r-project.org

7 http://amigo.geneontology.org/amigo

## Supplementary Material

FIGURE S1 Relationship between *CD96* and inflammatory activities in glioma.

FIGURE S2 Association between *CD96* expression and T cell NK and NK cell immune infiltrates. Red and blue text represents positive and negative associations with statistical significance (p-value < 0.05), respectively, and gray text represents non-significant correlations.

TABLE S1 GO biological process analysis results for top 50 genes most relevant to CD96 in the CGGA and TCGA databases.

TABLE S2 The most relevant immune genes to *CD96* in the CGGA and TCGA datasets. The red highlighted genes are involved in NK and T cell responses.

TABLE S3 Univariate and multivariate analyses of clinical prognostic parameters in CGGA and TCGA datasets.

## Abbreviations

AUC: area under the curve
CGGA: Chinese Glioma Genome Atlas
GBM: Glioblastomas
GO: gene ontology
GSVA: gene set variation analysis
IDH: Isocitrate dehydrogenase
OS: Overall survival
PD-L1: Programmed death-ligand 1
TCGA: The Cancer Genome Atlas dataset
TIM3: T cell immunoglobulin mucin-3
WHO: World Health Organization.
