# Corrigendum: Immune and Clinical Features of *CD96* Expression in Glioma by *in silico* Analysis

**DOI:** 10.3389/fbioe.2020.599544

**Published:** 2020-10-23

**Authors:** Qiang Zhang, Hua Zhong, Yinchun Fan, Qian Liu, Jiancheng Song, Shengtao Yao, Fang Cao

**Affiliations:** ^1^Department of Cerebrovascular Disease, Affiliated Hospital of Zunyi Medical University, Guizhou, China; ^2^College of Life Sciences, Wuhan University, Wuhan, China

**Keywords:** *CD96*, glioma, immune checkpoint, prognosis, immunotherapy

In the published article, there was an error in affiliation 1. Instead of “Department of Cerebrovascular Disease, The First Affiliated Hospital of Zunyi Medical University, Guizhou, China”, it should be “Department of Cerebrovascular Disease, Affiliated Hospital of Zunyi Medical University, Guizhou, China”.

The authors apologize for this error and state that this does not change the scientific conclusions of the article in any way. The original article has been updated.

