# Supplementary figures and images for: Immune and Clinical Features of CD96 Expression in Glioma by in silico Analysis
Source: Front Bioeng Biotechnol. 2020 Jun 30;8:592. doi: 10.3389/fbioe.2020.00592 (PMC7338376; doi:10.3389/fbioe.2020.00592)

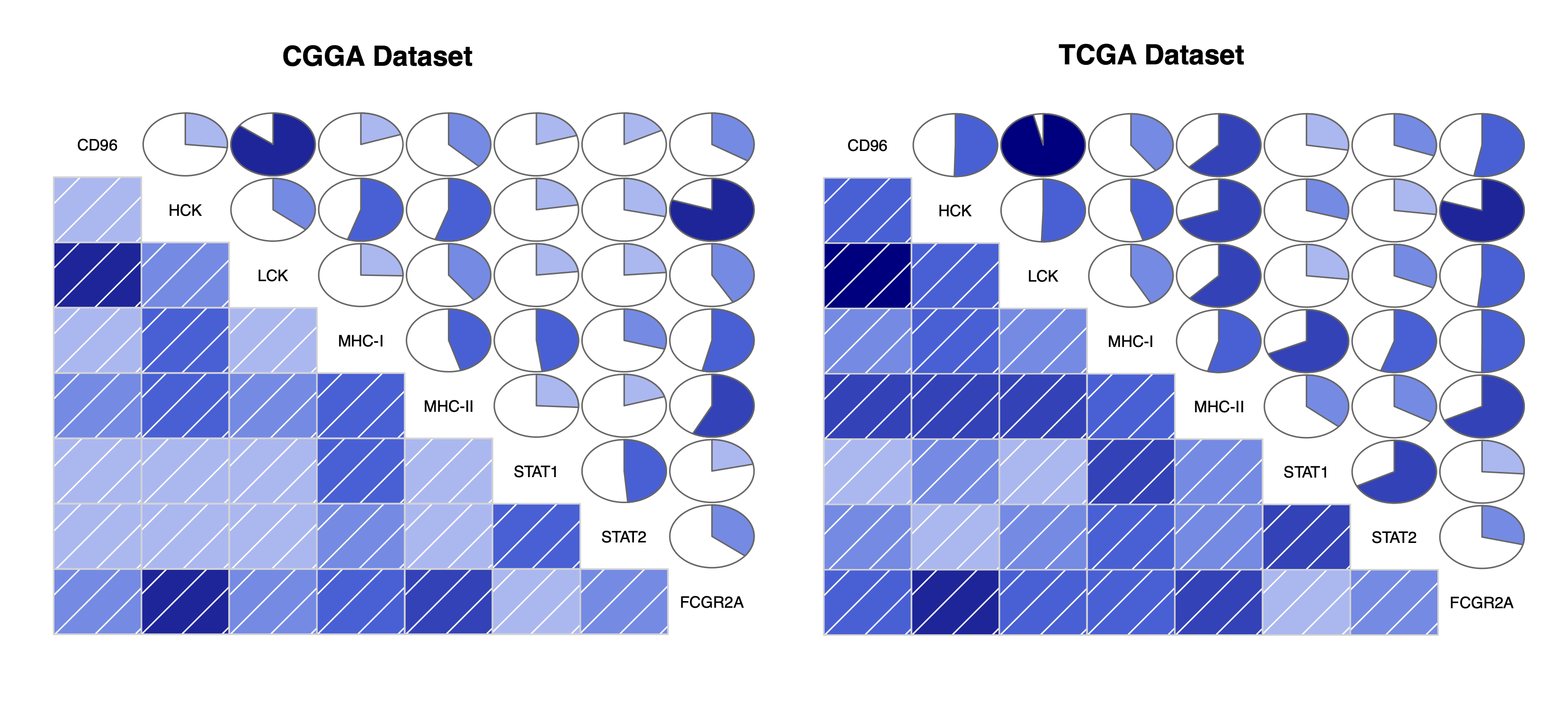

Supplement: FIGURE S1 — Relationship between CD96 and inflammatory activities in glioma. [file Image_1.JPEG]

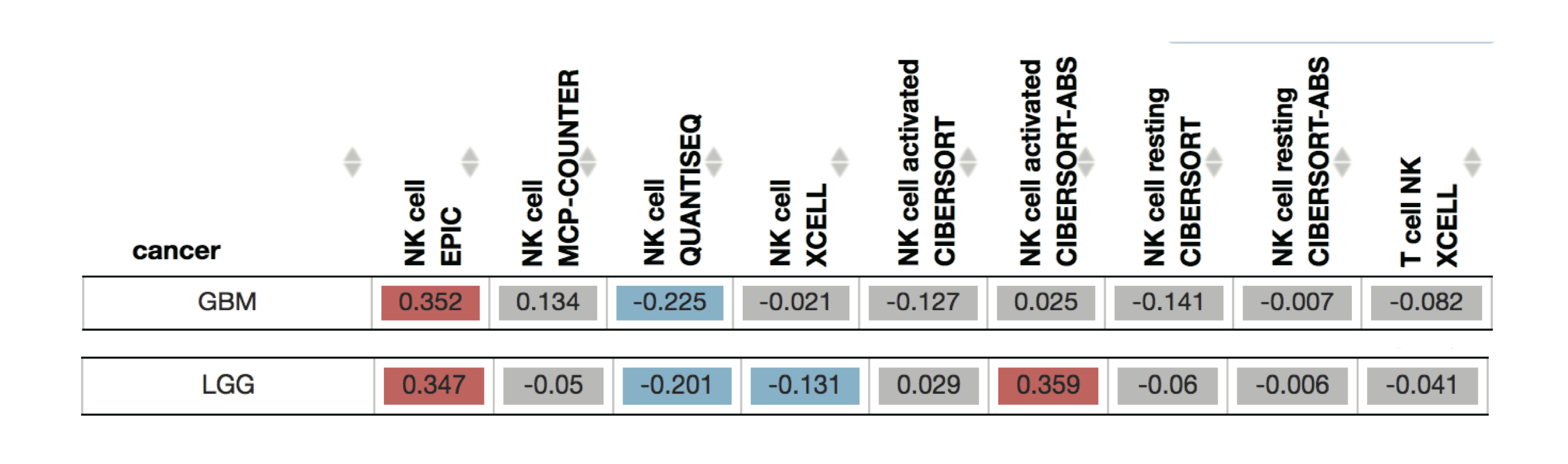

Supplement: FIGURE S2 — Association between CD96 expression and T cell NK and NK cell immune infiltrates. Red and blue text represents positive and negative associations with statistical significance (p-value < 0.05), respectively, and gray text represents non-significant correlations. [file Image_2.JPEG]
